# Dataset of molecular dynamics simulation trajectories of amino-acid solutions with various force fields, water models and modified force field parameters

**DOI:** 10.1016/j.dib.2020.105483

**Published:** 2020-04-08

**Authors:** Jiří Průša, Michal Cifra

**Affiliations:** a Institute of Photonics and Electronics of the Czech Academy of Sciences, Prague, Czech Republic; b University of Chemistry and Technology, Prague, Czech Republic

**Keywords:** Molecular dynamics, Force field, Amino acid, Dielectric spectroscopy, Biomolecules

## Abstract

We present molecular dynamics (MD) trajectories of water solutions of eight zwitterionic amino-acids (L- form) glycine (GLY), alanine (ALA), proline (PRO), threonine (THR), leucine (LEU), glutamine (GLN), histidine (HIS) and tyrosine (TYR) using various force field (OPLS-AA, Amber99ff-SB, GROMOS96 54a7, CHARMM19) and water model (SPC/E, TIP3P) combinations. Additionally, we present OPLS-AA molecular dynamics (MD) trajectories for alanine (ALA), leucine (LEU), glutamine (GLN), and tyrosine (TYR) varying the values of major force field parameters: charge on all amino acid atoms, bond length (all amino acid bonds), Lennard-Jones potential epsilon parameter and stiffness of bond angles. Our data enable to uncover sensitivity of molecular dynamics derived analysis to variation of force field and water models and force field parameters. This data set was used to understand the effect of molecular dynamics parameters on dielectric properties of amino acid solutions [1].

Specifications table

**Table:** 

Subject	Chemistry, Biology
Specific subject area	Computational Molecular biophysics
Type of data	Molecular Dynamics (MD) simulations
How data were acquired	Classical MD simulation in explicit solvent
Data format	Raw (molecular dynamics trajectories - compressed GROMACS trajectory files (.xtc))
Parameters for data collection	NVT ensemble at 300 K
Description of data collection	Data were obtained from molecular dynamics simulations ran on CESNET MetaCentrum virtual organisation of the Czech National Grid Organization with GROMACS software version 5.1.1
Data source location	Institution: Institute of Photonics and Electronics of the Czech Academy of Sciences City/Town/Region: Prague Country: Czechia
Data accessibility	Data is stored in a public repository Repository name: Zenodo Data identification number: DOI 10.5281/zenodo.3676936 Direct URL to data: http://dx.doi.org/10.5281/zenodo.3676936
Related research article	Authors: Jiří Průša ^1,2^, Michal Cifra ^1^ Title: Computational dielectric spectroscopy of amino acid solutions: dissecting the effects of force field parameters Journal: *Journal of Molecular Liquids* 10.1016/j.molliq.2020.112613

## Value of the data

• Data enable understanding of the influence of force field parameters and water models on the outcome from the generated molecular dynamics trajectories.
• Computational chemists, physical chemists, chemical biologists, bioelectromagnetic engineers and biophysicists can benefit from the data
• The data can be further used to obtain insights into how the basic properties of small molecules affect fundamental physical and chemical features of water-based solutions

## 1 Data

All raw data are available in the Zenodo repository under following folder structure. Standard (unmodified) force field simulations are in the folder FF_COMPARE. The simulations with changed parameters of the force field are in folder CHANGED_PAR. These two main folders with trajectories contain directories named by amino-acid three letter code. Within the amino-acid directories, the trajectories together with .pdb file of the initial system are placed in TRAJ directory and appropriate topologies in TOP directory.

### 1.1 Raw data from molecular dynamics using seven force field-water model combination

We provide trajectories for glycine (GLY), alanine (ALA), proline (PRO), threonine (THR), leucine (LEU), glutamine (GLN), histidine (HIS) and tyrosine (TYR) for seven force field-water model combinations.

The path MDP\md.mdp points to file with the settings of the molecular simulation runs.

The path for standard trajectories in xtc format is:

**FF_COMPARE**/AA-CODE/**TRAJ**/FF-NAME/WM-NAME/*.xtc

The path for the Initial coordinate files in .pdb format is:

**FF_COMPARE**/AA-CODE/**TRAJ**/FF-NAME/WM-NAME/*.pdb

The **bold text** in the file path is static and the rest is variable.

AA-CODE stands for “amino acid code”: GLY, ALA, PRO, THR, LEU, GLN, HIS, TYR

FF-NAME stands for “force field name”: OPLS, GROMACS, AMBER, CHARMM, CHARMM19

WM-NAME stands for “water model name”: SPCE*, TIP3P*

*where accessible

### 1.2 Raw data from molecular dynamics using modified force field parameters

We provide trajectories for alanine (ALA), leucine (LEU), glutamine (GLN), and tyrosine (TYR) for OPLS-AA/LL force field with changed force field parameters. We explored the range of the following parameters:

• Charge on all amino acid atoms (AC): -5%, -2.5%, 2.5%, 5%
• Bond length (all amino acid bonds) (BOND): -5%, -2.5%, 2.5%, 5%
• Lennard-Jones epsilon parameter (LJ): -5%, -2.5%, 2.5%, 5%
• Stiffness of angles and dihedral angles (DIAN): -10%, -5%, 5%, 10%

Here for each condition we run three independent simulations with different randomly assigned starting configuration of the molecules in the box and different initial velocity assigned to each atom.

The path of files obtained using force fields with modified parameters is:

**CHANGED_PAR**/AA-CODE/**TRAJ**/PARAMETER-CHANGED/DEGREEofCHANGE/*.xtc

The modified force fields are under

**CHANGED_PAR**/AA-CODE/**TOP**

The **bold text** in file path is static and rest is variable.

AA-CODE stands for “amino acid code”: ALA, LEU, GLN, TYR

PARAMETER-CHANGED stands for which force field parameter was modified: AC, BOND, LJ, DIAN

DEGREEofCHANGE stands for how much was the parameter modified: 0025*, 005*, 010*, m010*, m005*, m0025*

*where accessible

In the DEGREEofCHANGE, 005 means 5% and 0025 change of 2.5% the “m” character at the beginning of the directory name indicates negative change (m005 equals -5%).

### 1.3 Illustrative derived data

**Fig. 1:**
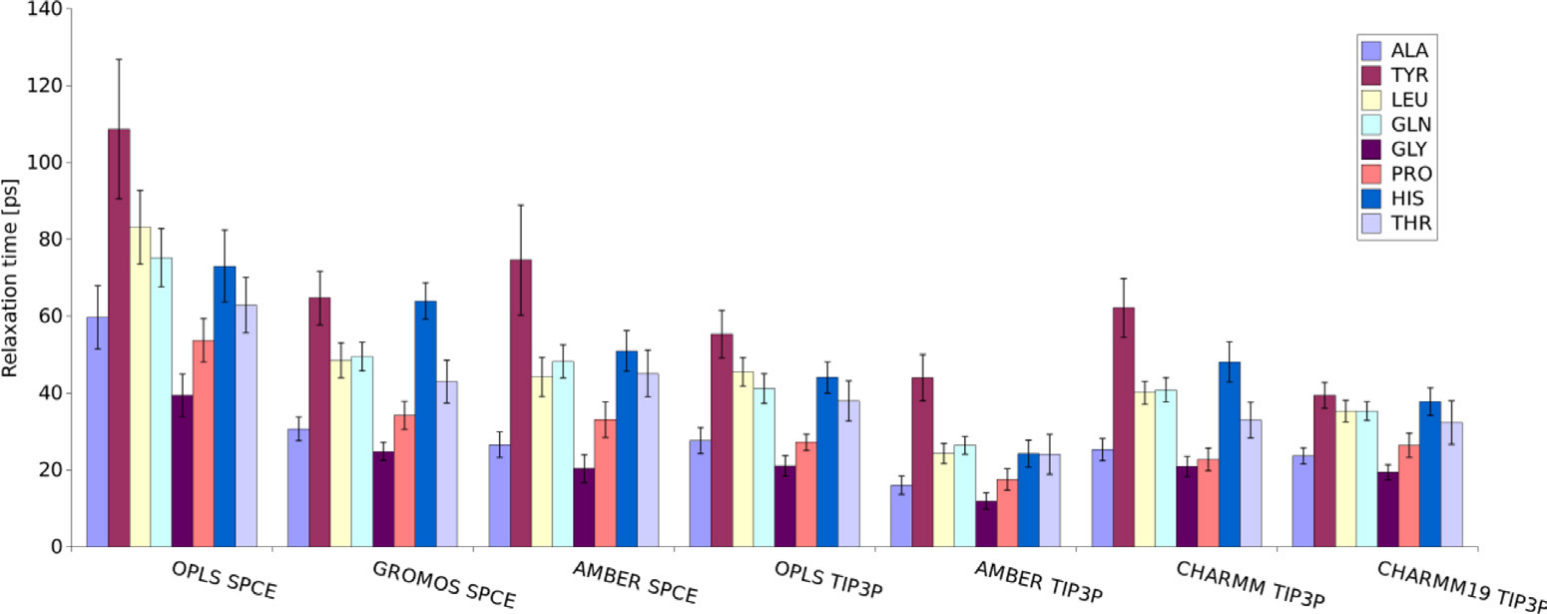
Rotational diffusion relaxation time of alanine (ALA), tyrosine (TYR), leucine (LEU), glutamine (GLN), glycine (GLY), proline (PRO), histidine (HIS), and threonine (THR) for various force field-water model combination. The graphs are derived from the raw data presented in this article. See [1] for more details.

Here we demonstrate the use of data we describe in our article. The trajectories from molecular dynamics simulation can be used to analyse variety of physical parameters of the solutions of amino acids. As an example, we focus here on a relaxation time (related to rotational diffusion time scale) of selected amino acids. Fig. 1 illustrates how relaxation time can vary based on the force field and water model used in the molecular dynamics simulations. See [1] for more details. Such data, when compared to experiment, enable better calibration of molecular dynamics parameters for prediction of electromagnetic (dielectric) response of biomolecules and biomolecular nanomachines.

## 2 Experimental design, materials, and methods

We performed all-atom MD simulations in explicit solvent of amino acids for various force fields and water models as well as using modified parameters of the OPLS force fields. The following combinations were explored:

**Table:** 

Force Field	Water Model
OPLS-AA/L	SPC/E
OPLS-AA/L	TIP3P
Amber99ff-SB	SPC/E
Amber99ff-SB	TIP3P
GROMOS96 54a7	SPC/E
CHARMM19	TIP3P
CHARMM27/CMAP	TIP3P

Each trajectory is 10 ns long (20 000 frames). 8 types of amino acids (GLY, ALA, PRO, THR, LEU, GLN, HIS, TYR) x 7 force fields-water model combinations x 10ns length of each trajectory = 560 ns total simulation length.

Additionally, we provide trajectories for alanine (ALA), leucine (LEU), glutamine (GLN), and tyrosine (TYR) for OPLS-AA/LL force field with modified force field parameters (charge on all amino acid atoms, bond length (all amino acid bonds), Lennard-Jones potential epsilon parameter, stiffness of angles and dihedral angles) as described in the Section 1.2.

Here for each condition we run three independent simulations. Therefore, 4 types of amino acids (ALA, LEU, GLN, TYR) x 4 force field modifications x 4 force field parameters x 10ns length of each trajectory x 3 repetitions = 1920 ns total simulation length.

### 2.1 Preparation of topologies

We used GROMOS, OPLS and CHARMM27 natively implemented in our GROMACS software version 5.1.1. Moreover, we have implemented CHARMM19 and AMBER parameters for zwitterionic amino acids [1].

### 2.2 MD simulations

All simulations were carried out with the same protocol. One molecule of the amino acid was placed in the middle of 32 × 32 × 32 Å^3^ water box, water molecules overlapping with amino acid were removed and the energy of the system was minimized by steepest descent algorithm. Thereafter we performed 10 ps equilibration part by pressure coupling with Parrinello-Rahman barostat [2] and increasing the temperature to 300K which was held constant by stochastic velocity rescaling algorithm [3]. Last part of the equilibration was 300 ps long and, same as in the main production run, NVT condition (300 K) was held by Nosé-Hoover thermostat [4] with the box dimensions inherited from NpT equilibration part. All Cα atoms were restrained from moving during whole equilibration part. The main production run was 10 ns long with all bond length constrained applying SHAKE algorithm [5]. Time step for numerical solution (leap-frog algorithm [6]) of Newton's equations of motions for both equilibration and simulation procedures was set to 2 fs. Evaluation of long-range electrostatics treated by Particle mesh Ewald (PME) method [7] the cut-off for van der Waals interaction and short-range electrostatic forces was set to 10 Å. The coordinates from trajectory were saved for every 0.5 ps for further analysis, thus yielding 20,000 frames from a single 10 ns trajectory.
